# Effect of 2% lidocaine continuous epidural infusion for thoracic or lumbar herpes-zosterrelated pain: Erratum

**DOI:** 10.1097/MD.0000000000013494

**Published:** 2018-11-21

In the article, “Effect of 2% lidocaine continuous epidural infusion for thoracic or lumbar herpes-zosterrelated pain”,^[1]^ which appeared in Volume 97, Issue 32 of *Medicine*, the authors’ affiliation appeared incorrectly and should be “Department of Pain Management, Wuhan No. 1 Hospital, Wuhan, Hubei Province, China.”
